# COMPSRA: a COMprehensive Platform for Small RNA-Seq data Analysis

**DOI:** 10.1038/s41598-020-61495-0

**Published:** 2020-03-12

**Authors:** Jiang Li, Alvin T. Kho, Robert P. Chase, Lorena Pantano, Leanna Farnam, Sami S. Amr, Kelan G. Tantisira

**Affiliations:** 1 The Channing Division of Network Medicine, Department of Medicine, Brigham & Women’s Hospital and Harvard Medical School, Boston, MA USA; 2 Boston Children’s Hospital, Boston, MA USA; 3 Harvard T.H. Chan School of Public Health, Boston, MA USA; 4 Partners Personalized Medicine, Boston, MA USA; 5 Division of Pulmonary and Critical Care Medicine, Department of Medicine, Brigham and Women’s Hospital, and Harvard Medical School, Boston, MA USA

**Keywords:** Data processing, Sequence annotation, Software

## Abstract

Small RNA-Seq is a common means to interrogate the small RNA’ome or the full spectrum of small RNAs (<200 nucleotide length) of a biological system. A pivotal problem in NGS based small RNA analysis is identifying and quantifying the small RNA’ome constituent components. For example, small RNAs in the circulatory system (circulating RNAs) are potential disease biomarkers and their function is being actively investigated. Most existing NGS data analysis tools focus on the microRNA component and a few other small RNA types like piRNA, snRNA and snoRNA. A comprehensive platform is needed to interrogate the full small RNA’ome, a prerequisite for down-stream data analysis. We present COMPSRA, a comprehensive modular stand-alone platform for identifying and quantifying small RNAs from small RNA sequencing data. COMPSRA contains prebuilt customizable standard RNA databases and sequence processing tools to enable turnkey basic small RNA analysis. We evaluated COMPSRA against comparable existing tools on small RNA sequencing data set from serum samples of 12 healthy human controls, and COMPSRA identified a greater diversity and abundance of small RNA molecules. COMPSRA is modular, stand-alone and integrates multiple customizable RNA databases and sequence processing tool and is distributed under the GNU General Public License free to non-commercial registered users at https://github.com/cougarlj/COMPSRA.

## Introduction

Small RNA sequencing (RNA-seq) technology was developed successfully based on special isolation methods and the RNA-seq technique, which facilitates the investigation of a comprehensive profile of small RNAs^1,2^. One of the most important applications is to quantify small RNAs in the circulatory system (circulating RNAs). The human circulatory system contains various types of RNA molecules, including fragmental mRNA, miRNA, piRNA, snRNA, snoRNA, and some other non-coding sequences^3,4^. Studies have shown the biomarker potential of circulating RNAs in cancer^5^, cardiovascular disease^6^, and asthma^7^. Moreover, other types of DNA and RNA fragments discovered in the human circulating system have been implicated as potential causes of chronic disease^8,9^.

In anticipation of a continued growing number of circulating RNAs studies, a comprehensive and stable platform is needed to identify the RNA classification, RNA read counts, differential expression between case and control samples, including both human and non-human (e.g. microbiome) small RNAs (<200 nucleotide length). Previous efforts to characterize small RNAs have focused primarily on microRNAs (miRNAs). For instance, sRNAnalyzer is a comprehensive and customizable pipeline for the small RNA-seq data centred on microRNA (miRNA) profiling^10^. sRNAtoolbox is a web-based small RNA research toolkit^11^ and SeqCluster has started to focus on non-miRNAs by comparing the sequence similarity^12^. Some efforts have begun to characterize the full spectrum of small RNAs of a biological system (the small RNA’ome), such as Oasis2, miRMaster and exceRpt^13–15^. Oasis2 and miRMaster are web servers for small RNA-seq data analysis. ExceRpt, maintained by the Extracellular RNA Communication Consortium (ERCC), is an extensive and commonly used web-based pipeline for extra-cellular RNA profiling. Oasis2 contains the pathogen detection module to detect the potential pathogenic infections or contaminations. miRMaster also permits the detection of potential exogenous miRNAs, but couldn’t handle case and control samples. ExceRpt provides few microbiome annotations. All the tools need users to upload the original sequencing files, which is un-workable for big data.

To profile intracellular and extracellular small RNA’omes through the small RNA-seq data, we built a comprehensive platform COMPSRA to identify and quantify diverse RNA molecule types, including miRNA, piRNA, snRNA, snoRNA, tRNA, circRNA and the fragmental microbial RNA. COMPSRA is built using Java and works as stand-alone providing detailed annotation for each type of small RNAs including microbial constituents. It currently uses STAR^16^ and BLAST^17^ for alignment and sequence comparison. It takes FASTQ file as inputs and outputs the counts profile of each type of RNA molecule type per FASTQ file (typically representing a sample). When case and control files are marked, COMPSRA can perform a differential expression analysis with the p value from the Mann-Whitney U test as default.

## Methods

**Figure 1:**
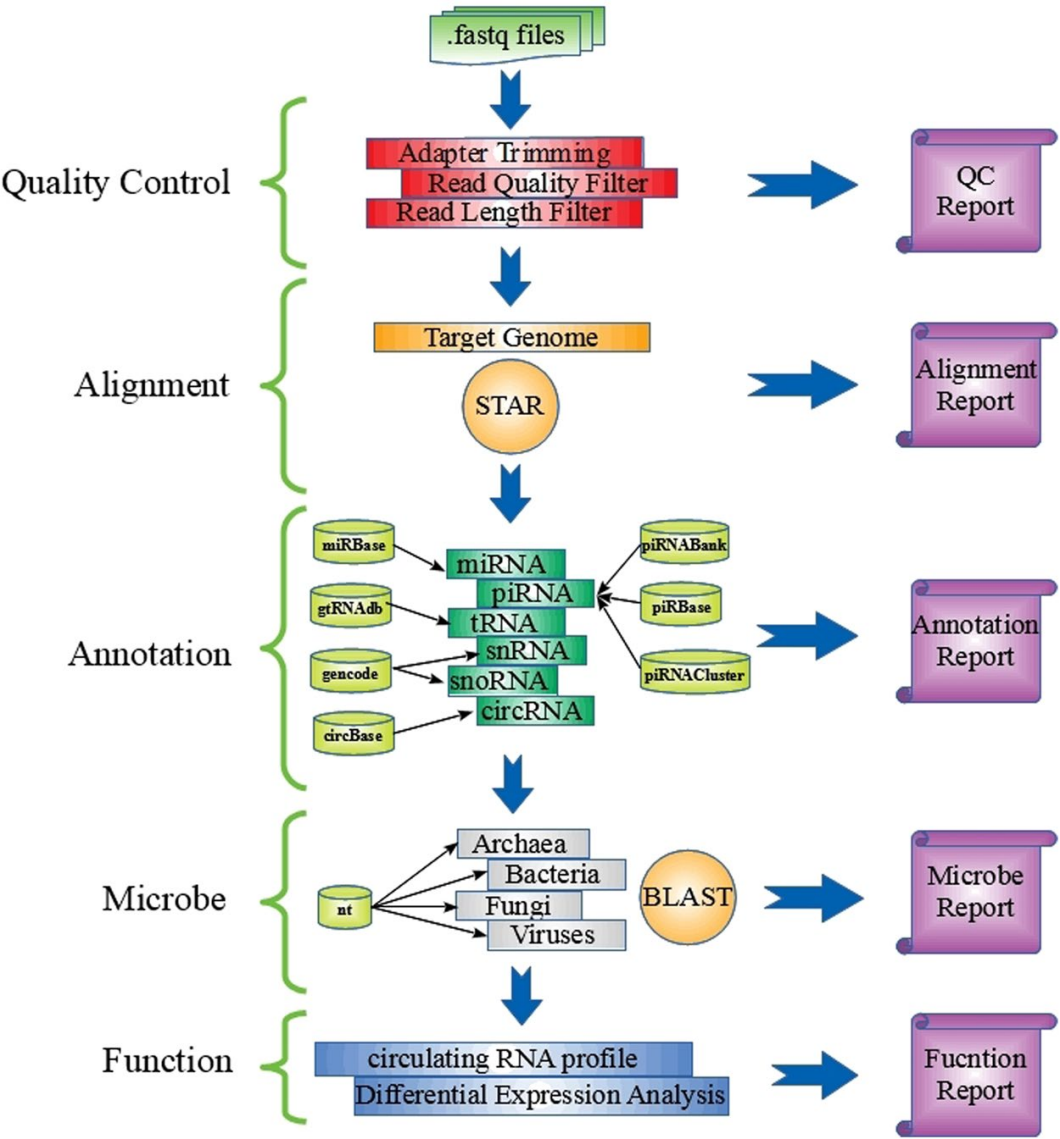
The structure of COMPSRA platform. COMPSRA is a comprehensive platform for circulating RNA analysis.

COMPSRA is built using Java and composed of five functionally independent and customizable modules: Quality Control (QC), Alignment, Annotation, Microbe and Function (see Fig. 1). Users can run all the modules as an integrated pipeline or just use certain modules. Each module is independent and it can run with appropriate inputs and parameters without having to go through earlier modules all at one time. Since COMPSRA is a stand-alone platform, it can be installed in any desktop or server, which maximizes data security and bypasses time/effort transferring data offsite that web-based tools need.

### Quality control (QC) module

FASTQ files from the small RNA-seq of biological samples are the default input. First, the adapter portions of a read are trimmed along with any randomized bases at ligation junctions that are produced by some small RNA-seq kits (e.g., NEXTflexTM Small RNA-Seq kit)^18^. The read quality of the remaining sequence is evaluated using its corresponding PHRED score. Poor quality reads (average PHRED ≤ 20) are removed according to quality control parameter set in the command line (−rr 20). The bases with bad quality (PHRED ≤ 20) in the head and tail ends of the read can also be removed with the related parameters (−rh 20 −rt 20). Users can specify qualified reads of specific length intervals for input into subsequent modules.

### Alignment module

COMPSRA uses STAR v2.5.3a^16^ as its default RNA sequence aligner and it will be updated with the latest version of STAR in the further release. Qualified reads from the QC module output are first mapped to the target genome (e.g., hg19/hg38), and then aligned reads are quantified and annotated in the Annotation Module. Reads that could not be mapped to the human genome are saved into a FASTA file for input into the Microbe Module. COMPSRA uses local type of read ends alignment because of the existence of miRNA isoforms (isomiRs)^19^. In order to make the alignment more accurate, only one mismatch is allowed in the default alignment parameter. For more detailed settings, users can refer to the online manual.

There are two scenarios where multi-aligned reads may exist when aligned against a reference genome. First, one small RNA read could be aligned to multiple distinct genomic locations. For example, the miRNA hsa-miR-1302 can derive from 11 potential pre-miRNAs. In this scenario, COMPSRA will only count once with the multi-aligned read. Second, two or more distinct small RNAs could have overlapping sequences. For example, miRNA has-let-7a-5p (**UGAGGUAGUAGGUUGUAUAGUU**) and piRNA has_piR_008113 (**UGAGGUAGUAGGUUGUAUAGUU**UUAGGGUC) have significant sequence overlaps. In this case, if the read can map to both, each small RNA will be assigned with one count.

### Annotation module

COMPSRA currently uses several different (and expandable) small RNA reference databases for annotating human genome mapped reads: miRBase^20^ for miRNA; piRNABank^21^, piRBase^22^ and piRNACluster^23^ for piRNA; gtRNAdb^24^ for tRNA; GENCODE release 27^25^ for snRNA and snoRNA; circBase^26^ for circular RNA. To harmonize the different reference human genome versions (hg18/hg19/hg38) in these databases, we use an automatic LiftOver created by the UCSC Genome Browser Group. All the databases used are pre-built to enable speedy annotation. For each RNA molecule, COMPSRA provides both the read count and indicates the database items that support its annotation. Using the command line parameter (-abam), COMPSRA will output all the reads that are annotated to a specific type of RNAs. COMPSRA still supports the annotation of small RNAs in the mouse genome and more species will be added in the latest release.

The annotation depends on the degree of read support (DRS), which was measured by the overlap between the gene locations and read mapped coordinates. We define a ratio ${R}_{olp}=\frac{{L}_{olp}}{{L}_{read}}$ to describe DRS, where *L*_*olp*_ denotes the number of overlapped bases and *L*_*read*_ denotes the number of bases of the target read. In COMPSRA, the default *R*_*olp*_ equals to 1 (-ann_overlap 1), which means the read should lie completely within the annotation.

### Microbe module (optional)

The qualified reads that were not mapped to the human genome in the Alignment Module are aligned to the nucleotide (nt) database^27^ from UCSC using BLAST. Because of the homology between species, one read may be aligned to many species and COMPSRA will list all the potential taxa with read count according to the phylogenetic tree as default. The four major microbial taxons archaea, bacteria, fungi and viruses are supported. To optimize processing the BLAST results, a fast accessing and parsing text algorithm is used^28^.

### Function module

The read count of each RNA molecule that is identified in the Annotation Module is outputted as a tab-delimited text file according to RNA type. With more than one sample FASTQ file inputs, the output are further aggregated into a data matrix of RNA molecules as rows and samples as columns showing the read counts of an RNA molecule across different samples. The default normalization method is Count-per-Million (CpM), which normalizes each sample library size into one million reads. The user can mark each sample FASTQ file column as either a case or a control in the command line, and perform a case versus control differential expression analysis for each RNA molecule using the Mann-Whitney rank sum test (Wilcoxon Rank Sum Test) as the default statistical test.

## Results and Discussion

We processed small RNA-seq FASTQ files from the serum of 12 healthy human subjects in a performance study through COMPSRA to evaluate its performance on diverse types of RNA molecules, and compare it to a previously published web-based pipeline exceRpt^15^. Serum samples were prepared using NEXTflex Small RNA Kit and sequenced through the Illumina platform.

We run COMPSRA on server with 30 GB RAM and set the same parameters as exceRpt. COMPSRA takes ~10 minutes per sample, but exceRpt will cost ~20 minutes for each sample (see Supplementary Fig. S1). The reason may be that COMPSRA maps raw reads to the genome directly and employs lots of pre-built database for annotation. If the microbiome module is required, more processing time will be cost, which depends on how many reads left to map to the microbial genomes and the total size of each kind of microbial genomes. For the 12 test samples, the estimated time on average is 3.5 hours, including viruses, archaea, bacteria and fungi (see Supplementary Table S1).

The output files of each type of RNAs contain four columns: DB (databases used for annotation), Name (name of the RNA), ID (general id of the RNA) and Count (counts of reads). A summary count file including all samples can be obtained from the function module (-fun_merge).

**Figure 2:**
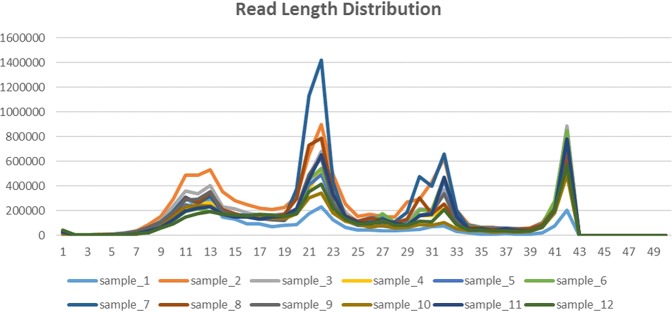
The read length distribution of 12 serum testing samples.

The read length distribution of 12 serum samples was described in Fig. 2. The length of raw reads was 50 nt and after trimming adapters and 4 random bases at both 5′ and 3′ ends, the read length varied from 0 nt to 42 nt. In general, without size selection at the library preparation stage, each read length distribution of one sample has 4 peaks. The miRNAs should be located around the main peak at 22 nt according to their structure characteristic. The piRNAs were distributed around 30 nt and the 32 nt peak represents the Y4-RNA (Ro60-associated Y4) and some tRNA fragments^29^. The 42 nt (or trimmed maximum read length in this study) might represent snRNAs, mRNA fragments and microbial RNAs. The snoRNA was overlapped with miRNA in a great measure. In addition, there were still large part of short RNA fragment around 12 nt, which may come from some RNA degradation products or even some unknown RNA classes.

**Figure 3:**
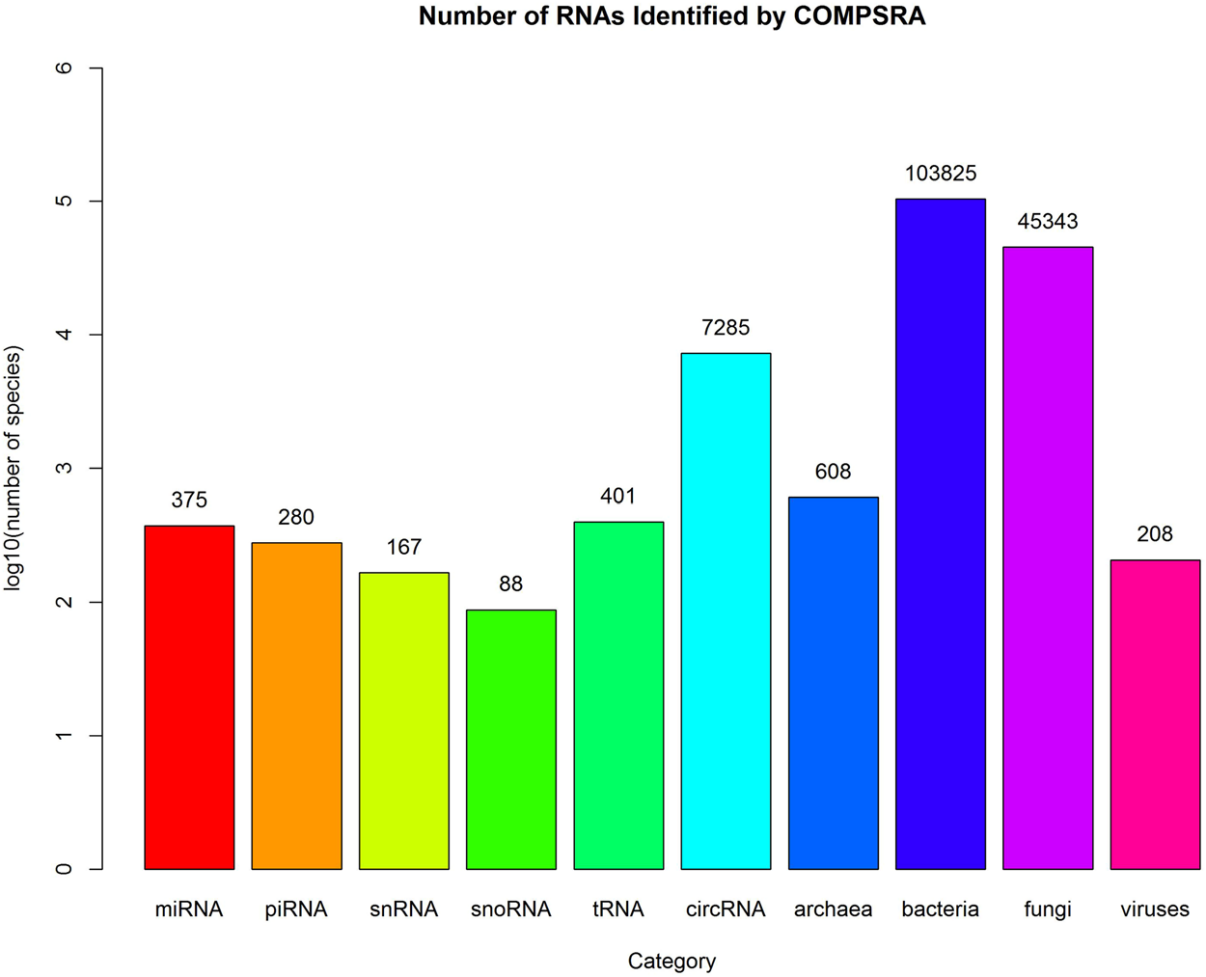
Number of RNAs Identified by COMPSRA through 12 serum samples.

COMPSRA identified diverse types of RNA molecules in this study including miRNAs, piRNAs, snRNAs, snoRNAs, tRNAs, circRNAs and RNAs in microbes (see Fig. 3). We used a read count threshold of 5 to indicate that a RNA molecule was detected (≥5) or not (<5). In total, COMPSRA detected 375 miRNAs, 280 piRNAs, 167 snRNAs, 88 snoRNAs, 401 tRNAs and 7285 circRNAs, as well as 608 archaea, 103825 bacteria, 45343 fungi and 208 viruses. The tRNAs were marked with the tRNAscan-SE IDs which were based on tRNA genes^30^. 7285 circRNAs were identified, which was much higher than other small RNAs. It might be because that the total number of circRNAs in human genome is huge. According to the statistics in CIRCpedia (v2), the human genome v38 (hg38) may contain 183,943 circRNAs^31^. The species of microbe were still large in number, which may be caused by the cross species homology. If one sequence read aligned to multiple homologous species, COMPSRA will output all the species without bias.

**Figure 4:**
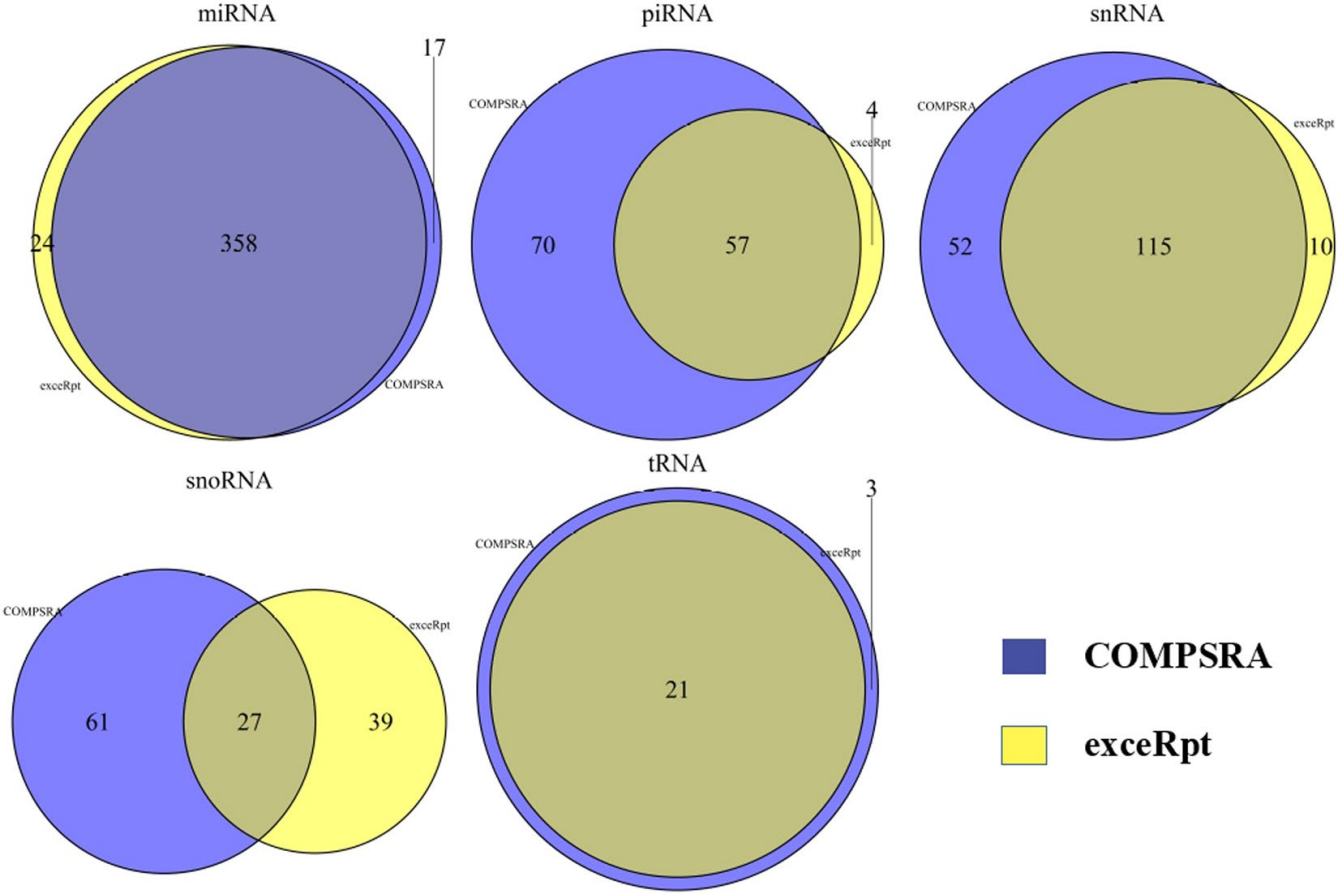
Summarize the comparison between COMPSRA and exceRpt.

Compared to exceRpt outputs of the same study data (See Fig. 4), COMPSRA generally shared a large proportion of commonly identified small RNAs with COMPSRA identifying more unique RNAs than exceRpt. For miRNAs, both COMPSRA and exceRpt identified 358 (90% of total miRNAs) miRNAs. Although exceRpt had 24 unique miRNAs, 18 (75%) of them were only detected in one sample. We listed the comparison of all the 12 samples between COMPSRA and exceRpt in Table 1. In each sample, the median counts of miRNAs from COMPSRA and exceRpt are nearly the same. COMPSRA and exceRpt had 27 common snoRNAs, among which 11 (41%) of them were detected only in one sample by COMPSRA and 15 (56%) of them detected only in one sample by exceRpt. COMPSRA had 61 unique snoRNAs, of which 41 (67%) snoRNAs existed only in one sample. However, exceRpt had 39 unique snoRNAs but 32 (82%) of them existed in one sample. snoRNAs were stable in circulation and they have been validated as biomarkers in some disease studies^32,33^. Compared with exceRpt, COMPSRA may have a more robust results in snoRNAs detection. In the comparison of tRNAs, we reclassified tRNAs according to the amino acid it carries as exceRpt did.

**Table 1:** miRNAs identified by COMPSRA and exceRpt among each sample.

	miRNA				
	COMPSRA(median)	exceRpt(median)	Overlap	COMPSRA_Unique	exceRpt_Unique
SAMPLE_1	64 (875.5)	66 (898)	63	1	3
SAMPLE_2	163 (491)	170 (492.5)	159	4	11
SAMPLE_3	174 (333)	176 (337)	164	10	12
SAMPLE_4	147 (545)	148 (557.5)	140	7	8
SAMPLE_5	123 (644)	123 (699)	117	6	6
SAMPLE_6	104 (723.5)	102 (754.5)	98	6	4
SAMPLE_7	240 (400.5)	249 (397)	234	6	15
SAMPLE_8	153 (607)	156 (568.5)	150	3	6
SAMPLE_9	195 (343)	200 (348)	187	8	13
SAMPLE_10	91 (767)	88 (747)	87	4	1
SAMPLE_11	125 (668)	126 (677.5)	121	4	5
SAMPLE_12	108 (671)	111 (703)	104	4	7

The comparisons of piRNAs, snRNAs, snoRNAs and tRNAs at the sample level were shown in Supplementary Tables S1–S4. COMPSRA can always identify more piRNAs than exceRpt (Table S1). The reason may be that COMPSRA use not only piRNABank database but also piRBase to annotate piRNAs. For snRNAs (Table S2), snoRNAs (Table S3) and tRNAs (Table S4), COMPSRA and exceRpt can detect a large set of common RNAs. There were more COMPSRA unique RNAs than than exceRpt unique RNAs, and a greater proportion of COMPSRA unique RNAs were detected in 2 or more samples than exceRpt unique RNAs. The median read count values from COMPSRA is usually larger than exceRpt. This could be because COMPSRA outputs the total read count for each RNA, while exceRpt normalizes the count by copy numbers. This will significantly decrease the count number when the RNA has more copies in the genome. In addition, exceRpt annotates the RNA types in order of priority (miRNA > tRNA > piRNA > snRNA and snoRNA > circRNA), so that when an aligned read has been annotated to a certain small RNA type, the read will not be annotated to the other types at a lower priority order. COMPSRA annotates an aligned read to all RNA types without an order of priority.

We downloaded the dataset SRP120169 from SRA database in NCBI as an extraneous reference. There were 42 small RNA-seq samples in the dataset and we run COMPSRA according to the parameters they set in the literature^34^. We take all the mature miRNAs in miRBase (v21) as a background and the miRNAs in the literature as benchmark, COMPSRA has a sensitivity of 85% and specificity of 87%. When comparing both the top 30% miRNAs identified, the sensitivity can reach 93% and specificity can reach 98% (see Supplementary Fig. S2).

We also check the 17 COMPSRA unique miRNAs and 24 exceRpt unique miRNAs above (Fig. 4A) in the SRP120169 data set. In total, 13 of the 17 COMPSRA unique miRNAs (76.47%) and 15 of the 24 exceRpt unique miRNAs (62.5%) exist in SRP120169. After filtering the read count less than 5, 13 miRNAs (76.47%) in COMPSRA and 11 miRNAs (45.83%) in exceRpt are identified, which means that the result from COMPSRA is more reliable and roust.

COMPSRA can align the reads that fail to map to the human genome with the microbial genomes and output the counts for each kinds of species. Because of the different parameters, databases and nomenclatures of species used, it is difficult to compare the results between tools in microbiome. In this paper, we attempt to run the 12 test samples on miRMaster and compared with COMPSRA among the top five commonly existed viruses. All the five viruses were also detected by COMPSRA. Pearson’s correlations were calculated across the 12 samples between COMPSRA and miRMaster (see Supplementary Table S6). The correlations of all the five species are greater than 0.8 and especially the correlation of phage phiX174 equals one, indicating a good consistency between miRMaster and COMPSRA. We also downloaded 9 samples from GSE59944, including 5 HIV-1 infected samples and 4 HIV-1 uninfected samples, to evaluate the performance of the microbe module in COMPSRA (see Supplementary Table S7). According to the results, HIV-1 was identified in all case samples but not in control samples and the read count of HIV-1 was always the top two in case samples, implying that COMPSRA has an excellent performance on the microbe module.

COMPSRA is a comprehensive modular stand-alone platform for the small RNA-seq data analysis. As a stand-alone platform, it bypasses data transfer effort/time/risk offsite that web-based tools need. Its modularity allows the user to run all modules together as a complete basic small RNA analysis pipeline or specific modules as needed. Its pre-built RNA databases and sequence read processing tools enable turnkey basic small RNA analysis from identification, quantification to basic differential analysis. These pre-built databases/tools are customizable and expandable.

## Supplementary information

Supplementary File.

## Data Availability

COMPSRA is distributed under the GNU General Public License free to non-commercial registered users at https://regepi.bwh.harvard.edu/circurna/ and the source code, as well as a detailed user manual and a sample test dataset, is available at https://github.com/cougarlj/COMPSRA.
