# Supplementary material for: COMPSRA: a COMprehensive Platform for Small RNA-Seq data Analysis
Source: Sci Rep. 2020 Mar 12;10:4552. doi: 10.1038/s41598-020-61495-0 (PMC7067867; doi:10.1038/s41598-020-61495-0)
Supplement: Supplementary file 1 — Supplementary File. [file 41598_2020_61495_MOESM1_ESM.pdf]

COMPSRA: a COMprehensive Platform for Small RNA-Seq data Analysis

Jiang Li[1,*], Alvin T. Kho[2], Robert P. Chase[1], Lorena Pantano[3], Leanna Farnam[1], Sami S. Amr[4], Kelan G. Tantisira[1, 5]

[1]The Channing Division of Network Medicine, Department of Medicine, Brigham & Women's Hospital and Harvard Medical School, Boston, MA, USA, [2]Boston Children's Hospital, Boston, MA, USA, [3]Harvard T.H.Chan School of Public Health, Boston, MA, USA, [4]Partners Personalized Medicine, Boston, MA, USA, [5]Division of Pulmonary and Critical Care Medicine, Department of Medicine, Brigham and Women's Hospital, and Harvard Medical School, Boston, MA, USA.

*To whom correspondence should be addressed.

Supplementary file1. Comparison of the runtime between exceRpt and COMPSRA.

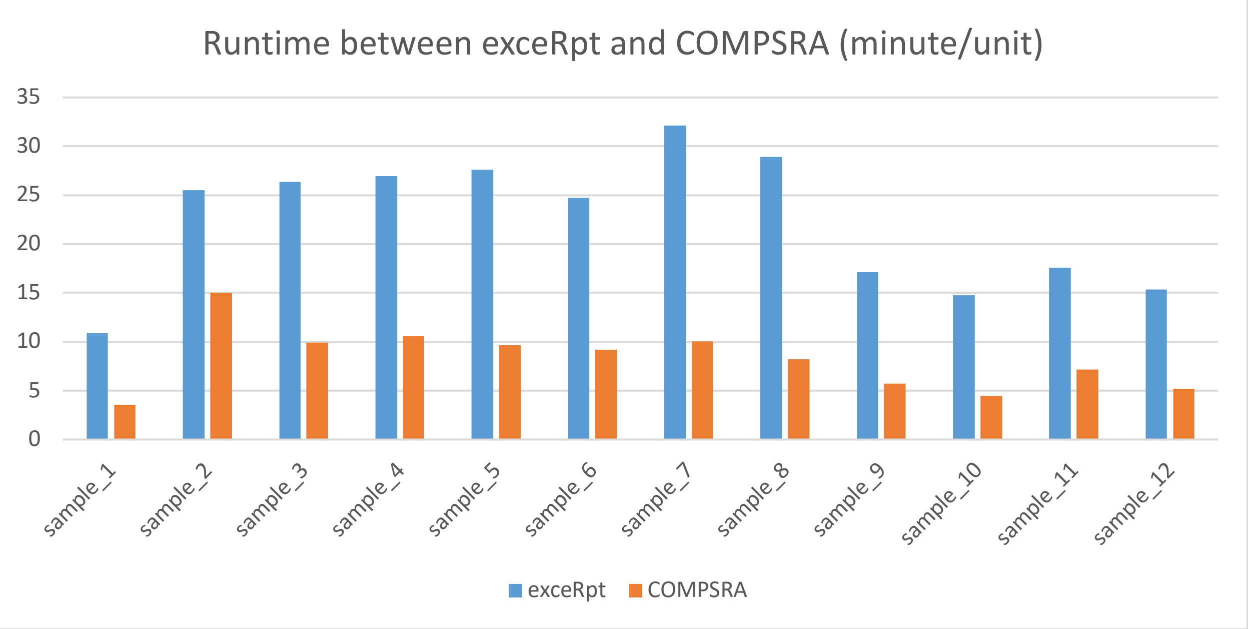

Figure S1. Runtime between exceRpt and COMPSRA. Generally COMPSRA run much faster than exceRpt. The reason may be that COMPSRA utilizes lots of pre-built database for annotation.

Supplementary file2. Estimated processing time on average of 12 samples according to different types of microbes.

Table S1. Estimated processing time on average of 12 samples according to different types of microbes.

Microbe	Time
Archaea	~12min
Bacteria	~1h20min
Fungi	~1h40min
Viruses	~16min

Supplementary file 3. The comparisons of piRNAs, snRNAs, snoRNAs and tRNAs at the sample level.

Table S2. piRNAs identified by COMPSRA and exceRpt among each sample.

	piRNA				
	COMPSRA (median)	exceRpt (median)	Overlap	COMPSRA_Unique	exceRpt_Unique
SAMPLE_1	35(686)	18(569.83)	16	19	2
SAMPLE_2	71(892)	26(2432.76)	26	45	0
SAMPLE_3	87(353)	34(1020.79)	33	54	1
SAMPLE_4	64(503)	25(1001.23)	25	39	0
SAMPLE_5	58(1134.5)	28(771.13)	27	31	1
SAMPLE_6	56(1915.5)	25(1771)	22	34	3
SAMPLE_7	84(675.5)	44(507.13)	42	42	2
SAMPLE_8	55(925)	26(1145.25)	26	29	0
SAMPLE_9	74(518.5)	34(592.42)	33	41	1
SAMPLE_10	47(681)	21(379)	21	26	0
SAMPLE_11	57(883)	30(789.5)	27	30	3
SAMPLE_12	55(455)	24(760.21)	24	31	0

Table S3. snRNAs identified by COMPSRA and exceRpt among each sample.

	snRNA				
	COMPSRA (median)	exceRpt (median)	Overlap	COMPSRA_Unique	exceRpt_Unique
SAMPLE_1	60(3974)	46(296.30)	43	17	3
SAMPLE_2	112(358.5)	82(82.84)	71	41	11
SAMPLE_3	123(130)	64(342.40)	59	64	5
SAMPLE_4	87(638)	57(295.49)	52	35	5
SAMPLE_5	43(744)	36(22.41)	32	11	4
SAMPLE_6	47(1600)	31(55.38)	28	19	3
SAMPLE_7	71(1098)	54(118.71)	48	23	6
SAMPLE_8	95(198)	87(22.21)	80	15	7
SAMPLE_9	100(117)	52(148.87)	48	52	4
SAMPLE_10	57(834)	34(45.11)	28	29	6
SAMPLE_11	59(1271)	53(117.92)	47	12	6
SAMPLE_12	55(703)	48(154.09)	45	10	3

Table S4. snoRNAs identified by COMPSRA and exceRpt among each sample.

	snoRNA				
	COMPSRA (median)	exceRpt (median)	Overlap	COMPSRA_Unique	exceRpt_Unique
SAMPLE_1	11(926)	7(145.8)	6	5	1
SAMPLE_2	14(998)	12(206)	8	6	4
SAMPLE_3	28(155)	12(203.1)	9	19	3
SAMPLE_4	21(320)	14(123.5)	12	9	2
SAMPLE_5	12(444)	8(43.5)	5	7	3
SAMPLE_6	17(430)	6(185.9)	5	12	1
SAMPLE_7	42(154)	23(112)	13	29	10
SAMPLE_8	12(849)	11(148.1)	8	4	3
SAMPLE_9	39(140)	39(6.49)	11	28	28
SAMPLE_10	3(184)	0(NA)	0	3	0
SAMPLE_11	9(550)	4(298)	1	8	3
SAMPLE_12	9(748)	6(53.9)	5	4	1

Table S5. tRNAs identified by COMPSRA and exceRpt among each sample.

	tRNA				
	COMPSRA (median)	exceRpt (median)	Overlap	COMPSRA_Unique	exceRpt_Unique
SAMPLE_1	13(11832)	10(1318)	10	3	0
SAMPLE_2	19(12996)	14(1168)	14	5	0
SAMPLE_3	22(7807.5)	18(696.5)	17	5	1
SAMPLE_4	20(8699.5)	13(1072)	13	7	0
SAMPLE_5	18(5860.5)	12(700.05)	12	6	0
SAMPLE_6	20(12415)	13(1700)	13	7	0
SAMPLE_7	21(10909)	18(908.5)	18	3	0
SAMPLE_8	18(7321.5)	13(539)	13	5	0
SAMPLE_9	23(5244)	18(796)	18	5	0
SAMPLE_10	14(10299)	11(742)	10	4	1
SAMPLE_11	19(7166)	13(962)	13	6	0
SAMPLE_12	18(10052)	12(684.5)	11	7	1

Supplementary file 4. Evaluation of COMPSRA in SRP120169 dataset.

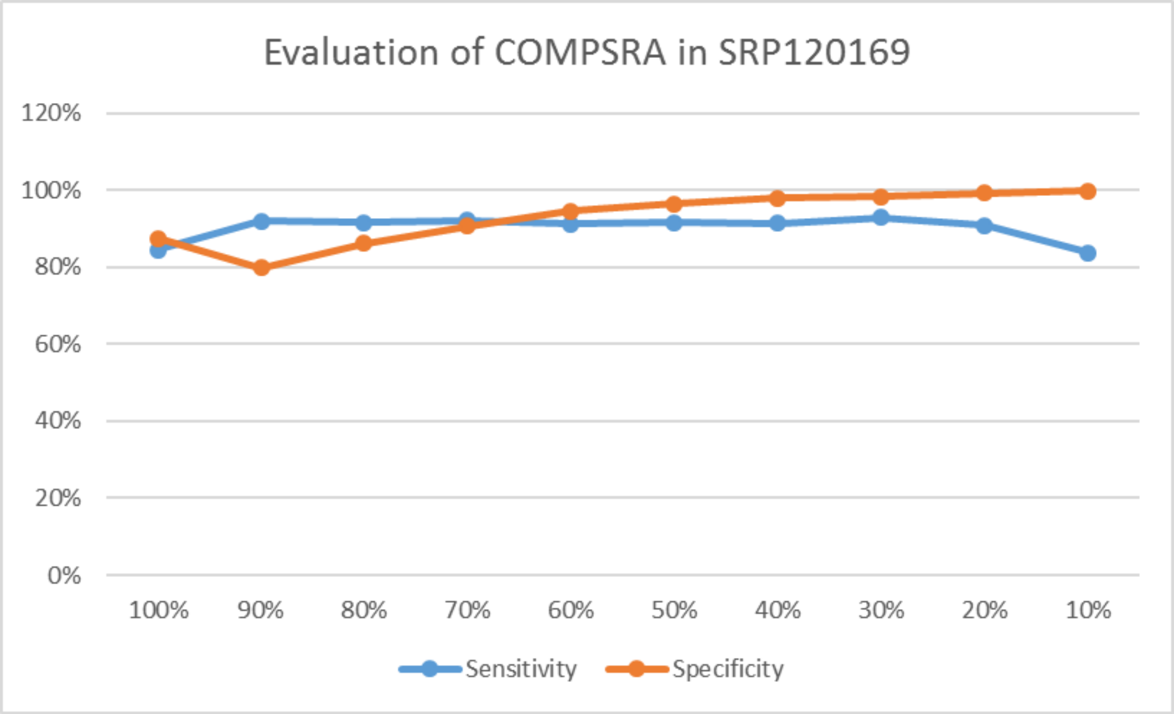

Figure S2. The sensitivity and specificity of COMPSRA in dataset SRP120169. The x-axis denotes the percentage of top miRNAs in both COMPSRA and SRP120169 literature results. The y-axis represents the percentage.

We take all the miRNAs in miRBase v21 as a background and compare the miRNAs identified by COMPSRA with the ones in the SRP120169 literature in light of the top percent of miRNAs. When comparing the total miRNAs, the sensitivity and specificity are 85% and 87% separately. The sensitivity reaches the highest value 93% in the top 30% of miRNAs dataset and then decreases to 84% in the top 10% of miRNAs dataset. The specificity decreases to 80% in the top 90% of miRNAs dataset, but increases to 100% gradually in the top 10% of miRNAs dataset.

Supplementary file 5. Top five commonly existed viruses identified by miRMaster and the correlations with COMPSRA.

Table S6. The Pearson's correlations of top five viruses.

Rank	Species	Correlation
1	Enterobacteria phage phiX174 sensu lato	1.00
2	Enterobacteria phage WA13	0.92
3	Enterobacteria phage St-1	0.92
4	Enterobacteria phage ID2 Moscow/ID/2001	0.81
5	Enterobacteria phage alpha3	0.87

Supplementary file 6. Test microbe module of COMPSRA in GSE59944 dataset.

Table S7. Detection of HIV-1 in GSE59944 dataset.

Sample ID	Cell Type	Treatment	(HIV) Read Count	Rank
GSM1462561	Cervical Epithelial Cell Line, HeLa derivative	TZM-bl **Uninfected** Day 3 Small RNA Deep Sequencing	NA	NA
GSM1462562	Cervical Epithelial Cell Line, HeLa derivative	TZM-bl BaL Day 3 Small RNA Deep Sequencing	1604	1
GSM1462565	CD4+ T-cell Line	C8166 **Uninfected** Day 3 Small RNA Deep Sequencing	NA	NA
GSM1462566	CD4+ T-cell Line	C8166 NL4-3 Day 3 Small RNA Deep Sequencing	16486	1
GSM1462567	CD4+ T-cells	CD4+ **Uninfected** Day 3 Small RNA Deep Sequencing	NA	NA
GSM1462568	CD4+ T-cells	CD4+ BaL Day 3 Small RNA Deep Sequencing	13621	2

GSM1462569	CD4+ T-cells	CD4+ NL4-3 Day 3 Small RNA Deep Sequencing	8339	1
GSM1462570	Monocyte-derived macrophage	Macrophage **Uninfected** Day 5 Small RNA Deep Sequencing	NA	NA
GSM1462571	Monocyte-derived macrophage	Macrophage ADA Day 27 Small RNA Deep Sequencing	109	2

We downloaded 9 small RNA deep sequencing samples from GSE59944 and run them through COMPSRA with the default parameters. The taxon with read count less than 50 was removed by rule of thumb. For HIV-1, we follow the taxon 11676(AIDS virus) [species] and the annotation of each sample was listed in Table S7. According to the result, HIV-1 was detected in all the case samples (infected) but not control samples (uninfected). The CD4+ T-cell may contain more HIV-1 than other cell types. Moreover, the read count of HIV-1 was always the top 2 of all the viruses detected in each sample. So, COMPSRA can identify the microbe accurately and has a good performance on the microbe module.
